# Effect of Graphene Oxide on Aging Properties of Polyurethane-SBS Modified Asphalt and Asphalt Mixture

**DOI:** 10.3390/polym14173496

**Published:** 2022-08-26

**Authors:** Shuai Li, Wenyuan Xu, Fengfa Zhang, He Wu, Qixin Ge

**Affiliations:** 1 College of Civil Engineering, Northeast Forestry University, Harbin 150040, China; 2 College of Civil Engineering, Heilongjiang Institute of Technology, Harbin 150050, China

**Keywords:** nano-modifier, modified asphalt mixes, thermal aging, OpenCV image technology

## Abstract

In order to clarify the effect of the new nano-material graphene oxide on the performance of Polyurethane-SBS modified asphalt and asphalt mixture under the effect of thermal aging, the cracking process of semicircular bending test (SCB) specimens was monitored in situ based on computer vision image processing technology (OpenCV), and the modified asphalt and the cracking characteristics of the modified asphalt and mixture were further analyzed by the tests of semicircular three-point bending and aggregate contact angle measurement. The test results showed that the thermal aging effect severely damaged the composite structure formed by the cross-linking effect of Polyurethane and SBS modifier in asphalt, which intensified the degradation of Polyurethane and SBS modifier and led to great changes in the rheological properties of asphalt after aging. However, the incorporation of the new nanomaterial Graphene oxide can slow down the degradation of Polyurethane and SBS modifiers and the change of asphalt cross-linked composite structure, making the anti-cracking and anti-aging properties of Graphene oxide-Polyurethane-SBS modified asphalt mixes better than those of Polyurethane-SBS modified asphalt mixes. Therefore, the new nano-material graphene oxide added to Polyurethane-SBS modified asphalt is meaningful and feasible. Graphene oxide-polyurethane-sbs composite modified asphalt, as a new nano-material modified asphalt, is stronger against the ultraviolet and light asphalt that is prone to aging. With regards to improving the application of road projects, the results are very promising.

## 1. Introduction

Asphalt mixtures are often subjected to thermal-oxidative aging during the construction process such as mixing, paving, rolling, and the service process of the road, thus making the asphalt mixture less resistant to cracking, which aggravates the occurrence of issues such as cracking and loosening of asphalt pavements [1,2,3,4]. This will inevitably affect the durability and service function of the road, directly leading to the shortening of the life cycle of the asphalt pavement and also increasing the road maintenance cost [5,6,7]. Therefore, clarifying the aging mechanism of asphalt, selecting suitable anti-aging additives, and effectively controlling the aging behavior of asphalt materials are essential to improve the quality of highway services and to enhance the sustainable development of transportation infrastructure construction [1,8].

Nanomaterials are materials that have at least one dimension in three-dimensional space with structural unit sizes within the range of 1 nm and 100 nm [9]. Due to the special properties of nanomaterials, the modification of asphalt using nanomaterials can significantly improve the viscoelasticity, fatigue resistance, high and low temperature properties, water damage resistance, and aging resistance of asphalt materials [10]. Therefore, modified asphalt using nanomaterials as modifiers has become a new research hotspot in the field of road traffic materials. Graphene oxide is a monoatomic layered structure formed by the oxidation of graphite that extends to tens of microns in lateral dimensions and has a large specific surface area and unique quasi-diametric layering ability, as well as an oxygen barrier [11,12,13,14]. Graphene oxide molecules can interact with aromatic and resin molecules in asphalt to improve the elastic response of the asphalt matrix and form a more stable molecular structure [15,16,17]. In addition, with the improvement of manufacturing technology, the cost of nanomaterials shows a decreasing trend [18], which makes it more possible to use graphene oxide as a new asphalt modification and to promote it on a large scale to improve the road performance of asphalt materials.

The preparation and characterization of composites based on SBS asphalt and polyurethane asphalt have been the subject of a great deal of research work in the past two to three decades, and the study of nano-modified asphalt has also made great progress. Zhang, Canlin et al. used cetyltrimethoxysilane organically modified layered double hydroxide (LDH) to modify SBS asphalt [19] and found that LDH could mitigate the aging damage to the performance and chemical structure of SBS modified asphalt and its resistance to aging was further enhanced. Liu, Shuai et al. used nano-organophosphate (A-Pal) to compound with star SBS to obtain modified asphalt [20], and after aging tests, it was found that A-Pal could reduce the thermal-oxidative decomposition of SBS and improve the aging resistance and fatigue resistance of SBS modified asphalt. Li Lihan and other researchers selected different binders such as acrylic resin, epoxy resin, and polyurethane to study their effects on the aging resistance of asphalt and cement pavements [21], and it was found that the acrylic resin binder system was better for the aging improvement of asphalt pavements. The aging resistance of polyether polyurethane concrete (PPC) for long-term use was evaluated by Xu, Shifa et al. After the Cantabro test, low temperature bending beam test, rutting test, splitting test under freeze-thaw cycles, and four-point bending fatigue test on aged PPC specimens, it was found that PPC has good aging resistance and can be used as a bridge deck pavement material for long-term use [22]. Li, Xian et al. investigated the modification of asphalt by adding graphene at nanoscale [23], and the results showed that graphene was uniformly dispersed as asphaltene with high surface energy and acted as a micelle nucleus in asphalt. Therefore, the incorporation of nanoscale graphene in asphalt can effectively improve the aging resistance, high temperature stability, and temperature sensitivity of asphalt. Cheraghian, Goshtasp et al. proposed to block the aging of asphalt by using engineered clay/fumed silica nanocomposites, and it was found that this composite effectively disrupts chemical oxidation and decomposition in the mixture and reduces the aging rate [24]. This cost-effective, simple, and scalable approach in warm mix asphalt mixtures helps to improve the sustainability and longevity of pavements and reduce greenhouse gas emissions.

The objective of this study was to evaluate the potential effect of Graphene oxide on the aging resistance of Polyurethane-SBS modified road asphalt mixtures. In detail, the microscopic morphology as well as the compositional, chemical, and anti-aging properties of Graphene oxide-Polyurethane-SBS modified asphalt and Polyurethane-SBS modified asphalt binder were carefully analyzed and supplemented with image processing techniques under computer vision to make the experimental results more intuitive. New insights are presented in further understanding the barrier properties of graphene oxide against Polyurethane-SBS modified asphalt aging. It is expected that this study will provide a reference for road design in areas where asphalt is prone to aging with higher UV and light exposure.

## 2. Materials and Methods

### 2.1. Materials

The procedures for the preparation of graphene oxide-polyurethane-SBS modified asphalt and Polyurethane-SBS modified asphalt and mixes were chosen based on previous studies by the authors, and the detailed steps are given in reference [7]. Graphene oxide (Qitaihe City, Heilongjiang Province, China; see Table 3 for specific indications; The oxygen content was 44.8%. The particle size D50(μ) was 30.22. BET surface area of >500 m2/g), Polyurethane (thermoplastic polyurethane particles (TPU), Germany), and styrene-butadiene-styrene triblock copolymer (SBSYH-792E thermoplastic styrene-butadiene rubber, Star, China; see Table 2 for specific indications) were used in this study. Mixture aggregate was produced in Yuchuan Quarry, Acheng District, Harbin, China (see Table 4 for classification). The asphalt content of the mixture was 4.3%. Based on previous experimental studies, the road performance of asphalt was found to be proportionally optimal when the Graphene oxide modifier dose was 0.5%; SBSYH-792E modifier dose was 4.5%; and TPU modifier dose was 5%. The composite modified asphalt has a degree of resistance to water damage, ductility, and chemical attack resistance. Therefore, the amount of graphene oxide modifier for the modified asphalt mentioned later was 0.5%; the amount of SBSYH-792E modifier was 4.5%; and the amount of TPU modifier was 5%.

### 2.2. Aging Process

Asphalt samples were maintained in a rotating film oven (RTFOT) at 163 °C for 85 min to prepare short-term aged asphalt according to the requirements in ASTM D1754 specifications. The asphalt samples were studied after long-term aging (temperature of 90 °C, pressure of 2.1 MPa, and time of 20 h) in a PAV according to the standard procedure of pressure aging vessel (PAV).

We prepared samples for six conditions. S1: unaged sample of Polyurethane-SBS modified asphalt, S2: unaged sample of Graphene oxide-Polyurethane-SBS modified asphalt; D1: short-term aged sample of Polyurethane-SBS modified asphalt, D2: short-term aged sample of Graphene oxide-Polyurethane-SBS modified asphalt; and C1: long-term aged sample of Polyurethane-SBS modified asphalt, C2 Graphene oxide-polyurethane-SBS modified asphalt long-term aging sample.

### 2.3. Test Methods

#### 2.3.1. Fluorescence Microscopy Test

Fluorescence microscopy tests were performed with an ortho-fluorescence microscope Axio Imager A2, manufactured by Zeiss, Germany. Before the test, the slide was put on the electric stove and heated slightly. An appropriate amount of asphalt was taken on the slide and allowed to flow naturally under the high temperature, while the coverslip was gently put on; the test was carried out once it reached room temperature. The wavelengths of the different ranges of light emitted by the polymer modifier under the irradiation of a fluorescent light source were observed to distinguish the polymer phase from the asphalt phase; thus, the true phase structure of the polymer phase in asphalt was observed [25].

#### 2.3.2. Video Optical Contact Angle Test

The contact angle test was conducted using an optical contact angle tester, model OCA20, manufactured by Dataphysics, Germany. The test sample was prepared by spraying asphalt on a slide before testing. The measurement was performed by the static drop method, where the prepared specimen was first placed on the bench, pressed with a spring plate, and the syringe was controlled to drop 5 μL of distilled water on the test sample, allowing the surface of the specimen to come into contact with the droplet, and then fitted using the supporting software to obtain the video optical contact angle. The strength of the asphalt hydrophobic properties determines to a certain extent the level of cracking resistance of the asphalt mixture.

#### 2.3.3. Semicircular Three-Point Bending Test (SCB)

**Figure 1:**
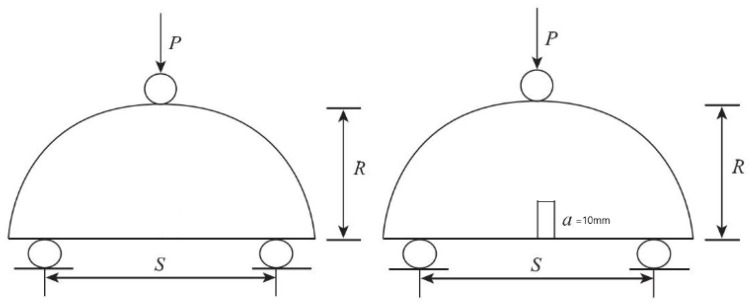
Schematic diagram of semicircular specimen.

In accordance with the JTGE20-2011 specification requirements, the semi-circular three-point bending test was conducted using standard Marshall specimens, and the diameter direction of the Marshall specimens was divided into two semi-circular specimens. In order to study the fracture performance of asphalt mixture, the low section of the semicircular specimen took two forms of 10 mm cut and no cut for control, as shown in Figure 1. Five specimens of each asphalt mixture were taken for parallel tests at 25 °C and the loading rate of the MTS testing machine was 0.5 mm/min. The information of load and displacement was obtained by MTS displacement sensors, and the test termination condition was complete fracture of the specimens [26,27].

To further evaluate the effect of Graphene oxide on the fracture resistance of Polyurethane-SBS modified asphalt mixes after aging, J-integral theory was introduced [28,29] to evaluate the ability of the mix to resist crack expansion by the area under the load-displacement curve, i.e., the fracture energy index.

#### 2.3.4. OpenCV Image Processing in SCB Testing

With the development of technology, the introduction of the field of artificial intelligence under computer vision into the field of road material science has become the trend of future development. OpenCV, an open source computer vision and machine learning library, is capable of performing the required image processing functions effectively [30,31]. In this study, the whole process of cracking of SCB specimens was recorded by OpenCV pixel addition and subtraction using Python programming language under Linux platform.

## 3. Results and Discussion

### 3.1. Analysis of Anti-Cracking Performance of Semicircular Bending Test

**Figure 2:**
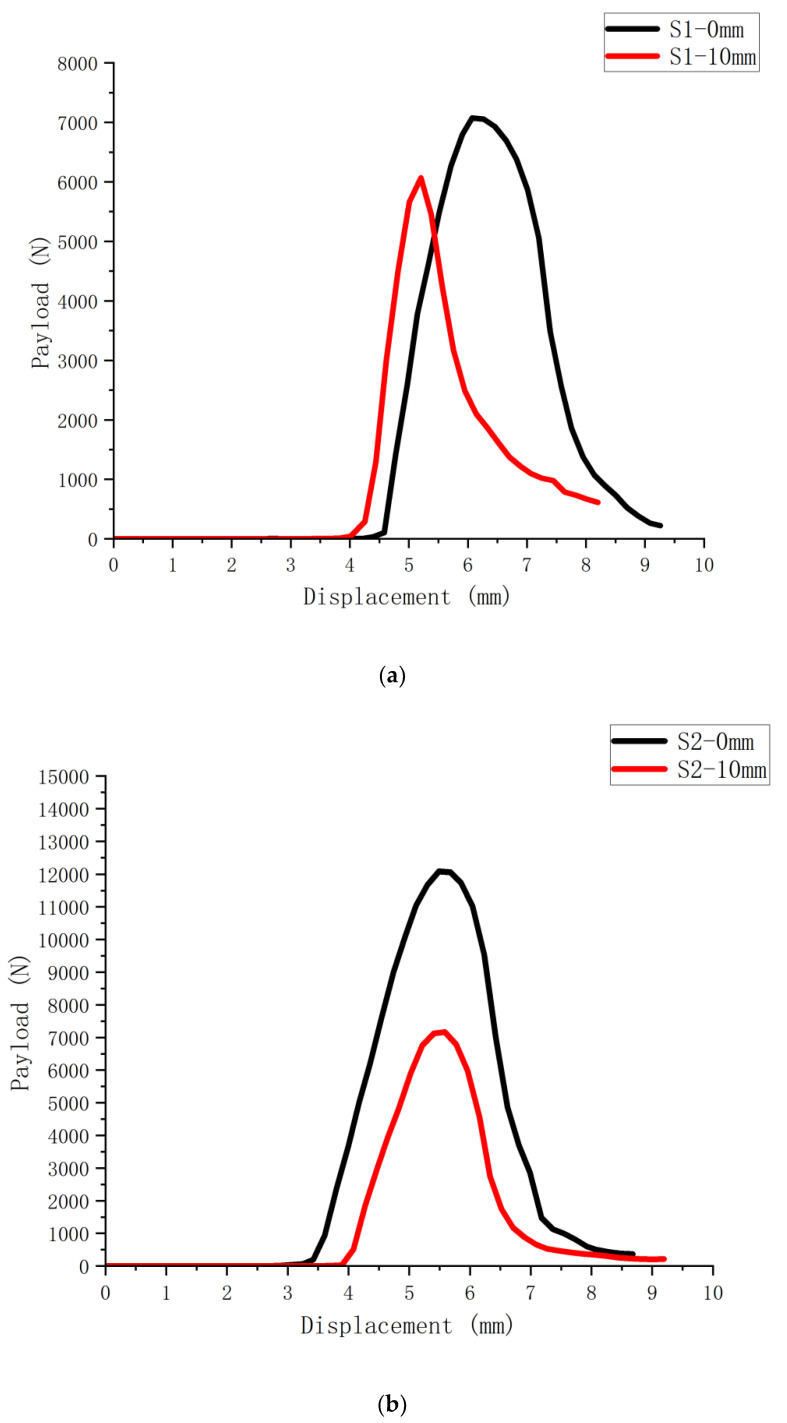
Load-displacement curves of unaged asphalt mixture SCB specimens: (**a**) S1 asphalt mixture SCB test curve; (**b**) S2 asphalt mixture SCB test curve.

**Figure 3:**
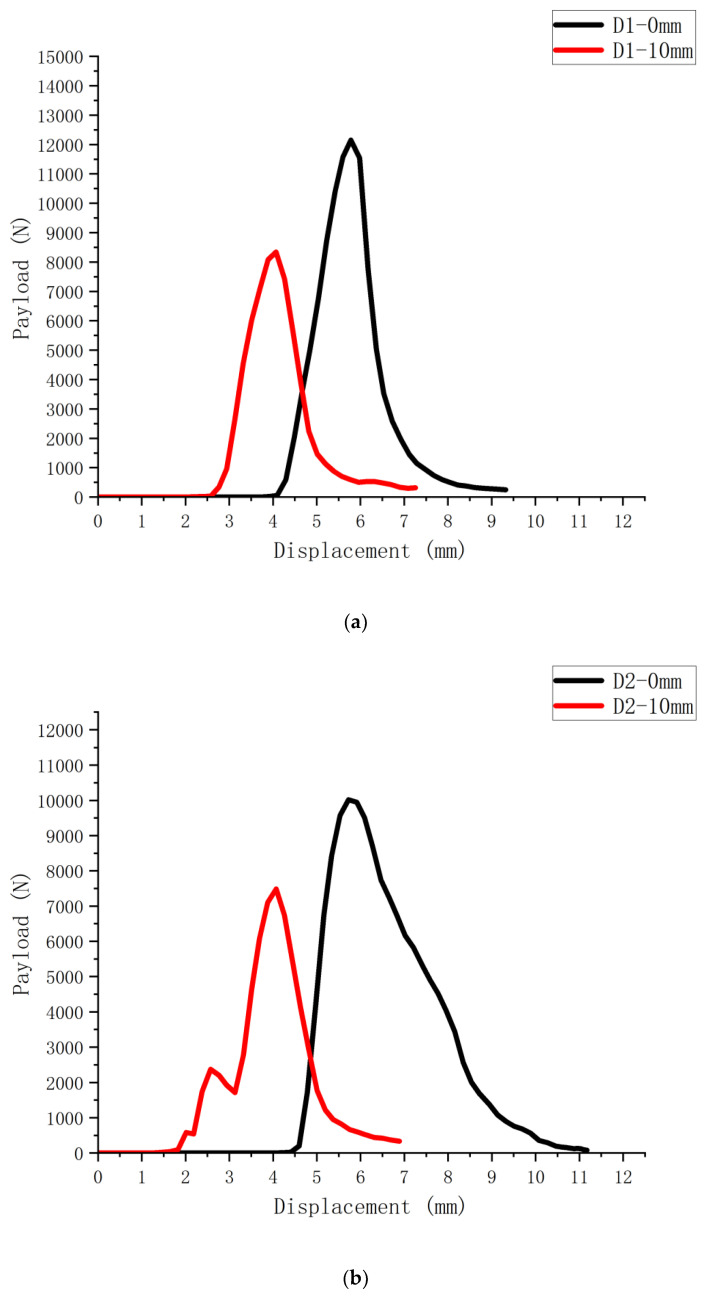
Load-displacement curves of short-term aged asphalt mixture SCB specimens: (**a**) D1 asphalt mixture SCB test curve; (**b**) D2 asphalt mixture SCB test curve.

**Figure 4:**
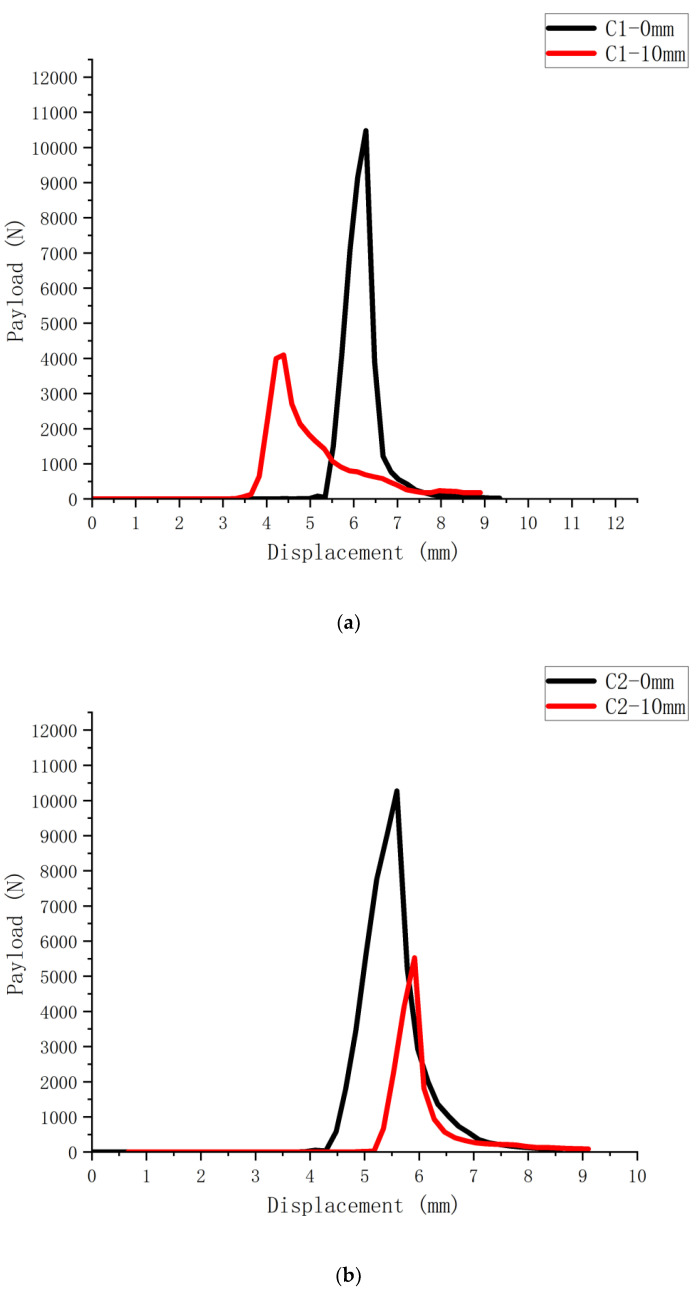
Load-displacement curves of long-term aged asphalt mixture SCB specimens:. (**a**) C1 asphalt mixture SCB test curve; (**b**) C2 asphalt mixture SCB test curve.

The load-displacement curves obtained by conducting bending and tensile damage tests on six SCB specimens with and without 10 mm cutouts are shown in Figure 2, Figure 3 and Figure 4 (S1-0 mm for the specimens without cutouts and S1-10 mm for the specimens with 10 mm cutouts). There is a significant difference between the maximum loads that the notched and uncut SCB specimens can withstand, with the notched specimens obviously having less strength and faster damage time than the uncut specimens.

According to the load-displacement curves of SCB specimens of different asphalt mixes, the fracture energy of the mixes can be obtained by bringing in the following Equation (1).

(1) $$G_{f}=\frac{1}{b\left(D-a\right)}\int_{0}^{u_{final}}P\left(u\right)du$$

where: *u_final_* is the displacement of the loading point corresponding to the damage of the specimen; *D* is the size of the specimen; and *a* is the depth of the crack.

To minimize the influence of external factors on the measurement reliability, the initial value is calculated at the time the pressure sensor produces the value.

**Figure 5:**
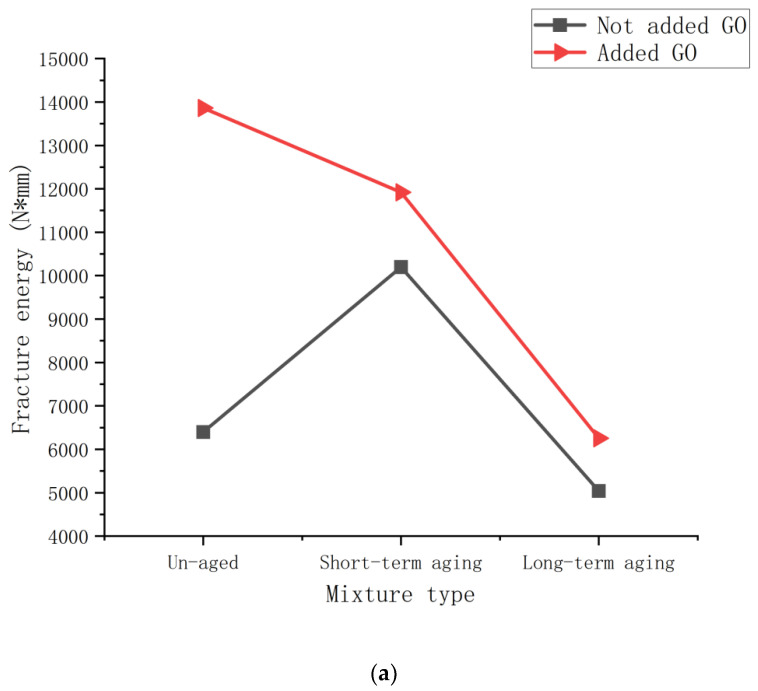

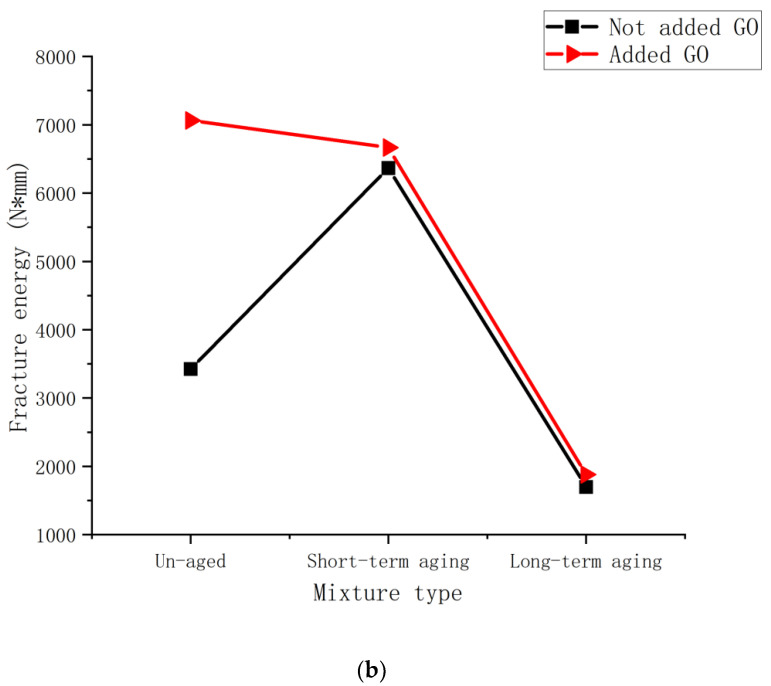
Fracture energy images of SCB specimens of asphalt mixes with different degrees of aging: (**a**) Fracture energy of uncut SCB specimens; (**b**) Fracture energy of cut SCB specimens.

Based on the obtained load-displacement curves and the above equations, the resulting fracture energy of SCB specimens with different mixes is calculated as shown in Figure 5.

A visual representation of the cracking performance of the asphalt mixture under different aging conditions can be seen in the figure. From Figure 5a,b, it can be seen that the incorporation of Graphene oxide can significantly improve the fracture resistance of the Polyurethane-SBS asphalt mixture, and it also has different degrees of effect on the polyurethane-SBS asphalt mixture after short-term and long-term aging, and this effect is especially obvious in the fracture energy diagram of the SCB specimens without notching.

With the deepening of aging, the fracture energy of Polyurethane-SBS asphalt mixture showed a form of increasing and then decreasing, indicating that after short-term aging, the cracking and damage resistance of Polyurethane-SBS asphalt mixture was substantially improved compared with the original asphalt. However, after long-term aging, the asphalt mixture becomes brittle and the possibility and speed of crack expansion becomes relatively large, i.e., long-term aging will accelerate the cracking process of asphalt mixture. The fracture energy of the Graphene oxide-Polyurethane-SBS asphalt mixture showed a tendency to decrease with the deepening of aging, indicating that the resistance to cracking and damage of the mixture became weaker with the deepening of the aging process.

The comparative trend in the figure shows that the incorporation of Graphene oxide increases the fracture resistance of SCB samples of Polyurethane-SBS asphalt mixture at any degree of aging under more or less the same conditions, and this improvement is particularly evident in the fracture energy diagram of the uncut SCB specimens. This indicates that both the cracking resistance and aging resistance of the Graphene oxide-Polyurethane-SBS modified asphalt mixture are better than those of the Polyurethane-SBS modified asphalt mixture. Therefore, it is meaningful and feasible to add the novel nanomaterial graphene oxide to Polyurethane-SBS modified asphalt as an anti-aging and anti-cracking additive, but Graphene oxide-Polyurethane-SBS modified asphalt mixture pavement needs proper maintenance work to avoid the degradation of its performance after cracking.

### 3.2. Opencv Images for SCB Test Cracking Performance Characterization

During the study of the cracking characteristics of the SCB test mix, the open-source computer vision package OpenCV [32,33], a Python programming language under Linux platform, was used to record the whole cracking process of the SCB specimens. The effect of Graphene oxide on the cracking resistance and aging resistance of Polyurethane-SBS modified asphalt mixture SCB specimens was characterized more intuitively through image processing and comparison of experimental data by computer vision.

During loading, the damage process of SCB specimens is mainly divided into the initial damage phase under fatigue loading, i.e., the crack initiation phase, and the damage destruction phase, i.e., the complete occurrence of cracks. In this study, we mainly used the OpenCV pixel addition and subtraction method to record the bending and tensile damage processes of six SCB specimens with and without 10 mm notches. The original unloaded stage of each specimen was used as the original sample, and OpenCV image processing was done with the crack initiation stage and the fully cracked stage to analyze the effect of different aging levels and the doping of Graphene oxide on the crack development.

In general, when using OpenCV image technology to captureg the cracking process of SCB specimens, the measurement error is less than 0.01 pixel, which can meet the requirements of asphalt measurement accuracy, and the dark asphalt mastic on the surface of asphalt mixture and light-colored aggregate sections can form a sharp contrast color that can be easily captured by OpenCV image technology.

**Figure 6:**
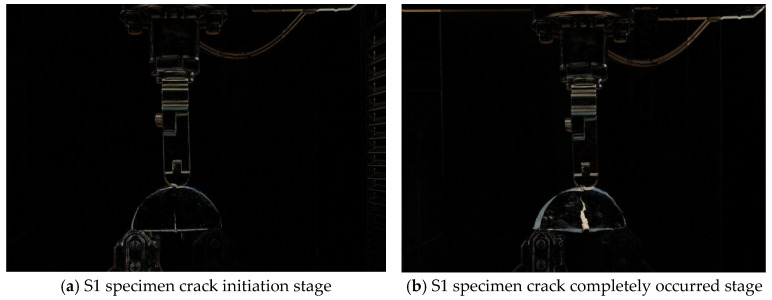

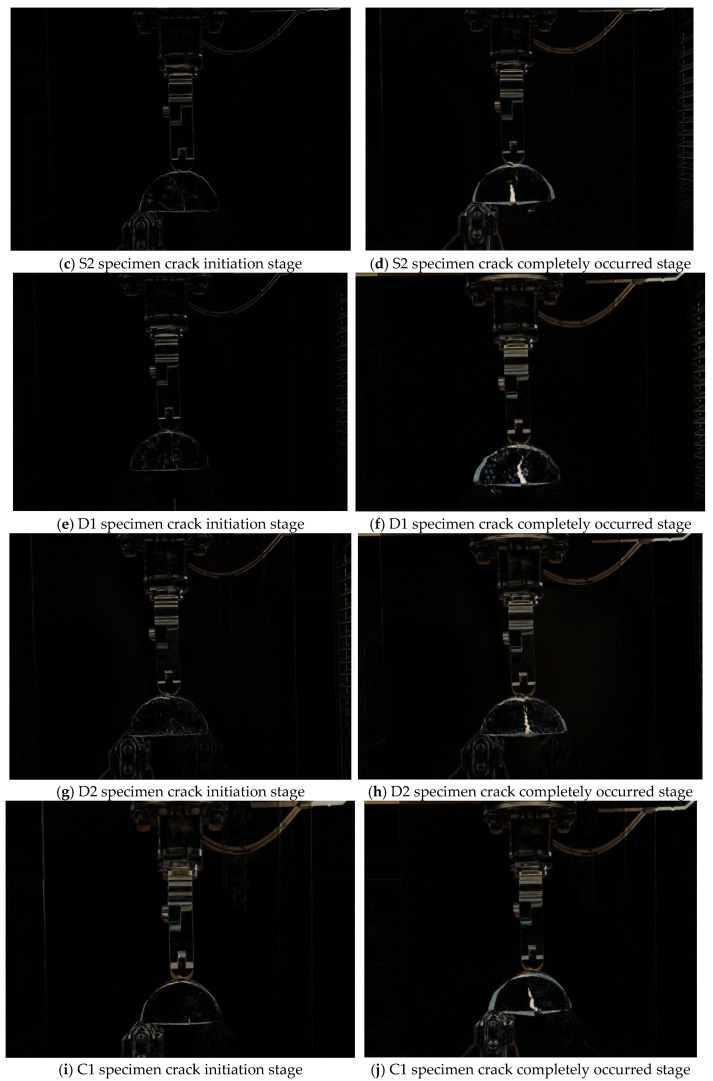

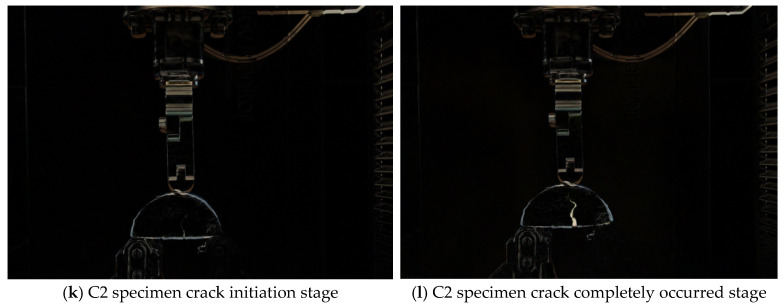
The damage process of the unopened SCB specimen.

**Figure 7:**
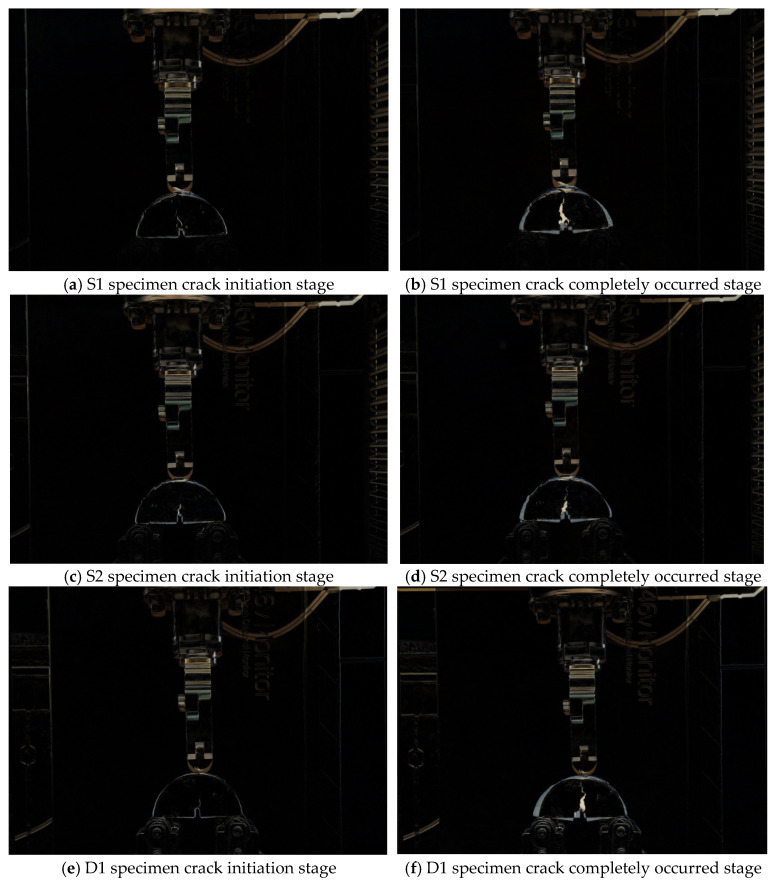

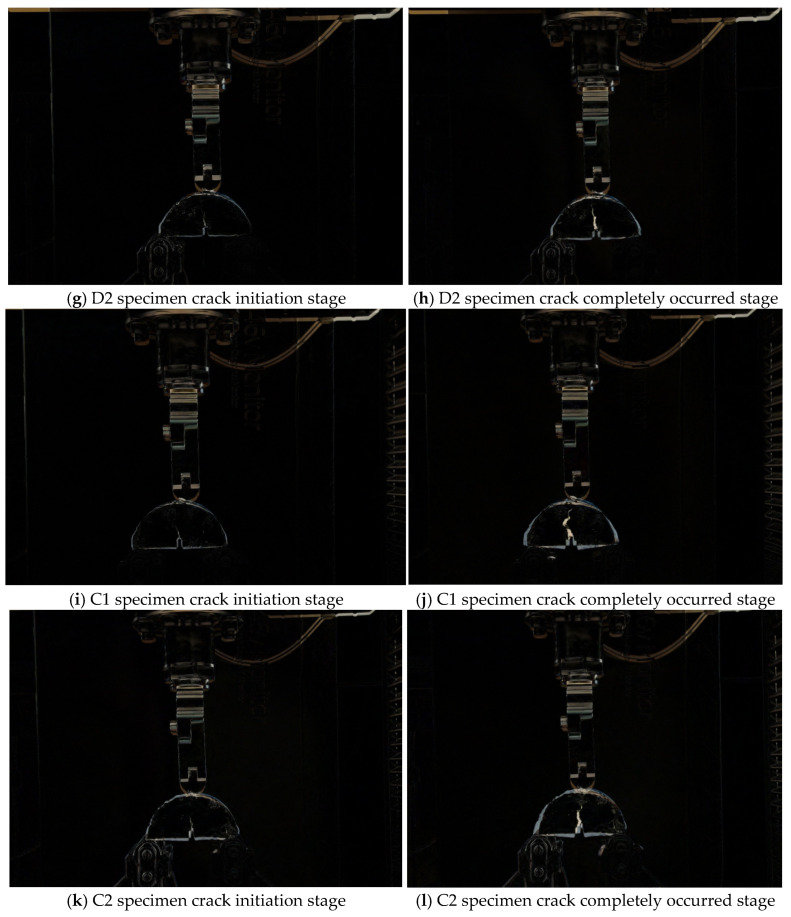
The damage process of SCB specimen with 10 mm opening.

The processing results are shown in Figure 6 and Figure 7. Figure 6 shows the loading damage process of six different SCB specimens without preset notches, and Figure 7 shows the loading damage process of six different SCB specimens with preset 10 mm notches. From the comparison in Figure 6, it can be seen that for the specimens without preset cuts, the depth of crack development and the degree of deformation of the specimens at the time of damage are smaller for the specimens with Graphene oxide addition than for the specimens without Graphene oxide addition when loaded to complete damage from the unaged to fully aged stage, and this result is in accordance with the conclusion drawn from the semicircular bending test. From the comparison in Figure 7, it can be seen that for the specimens with a preset 10 mm cut, the specimens with Graphene oxide added have a more similar depth of crack development than the specimens without Graphene oxide added when loaded to complete damage, but the degree of deformation is significantly smaller. It is obvious from the treatment result graph that the anti-aging performance and post-aging cracking resistance of the asphalt mixture doped with Graphene oxide are significantly better than that of the mixture without Graphene oxide. This means that the incorporation of Graphene oxide can greatly improve the aging resistance of asphalt mixes and the ability to resist cracking and deformation behavior after aging.

### 3.3. Contact Angle of Asphalt Binder

**Figure 8:**
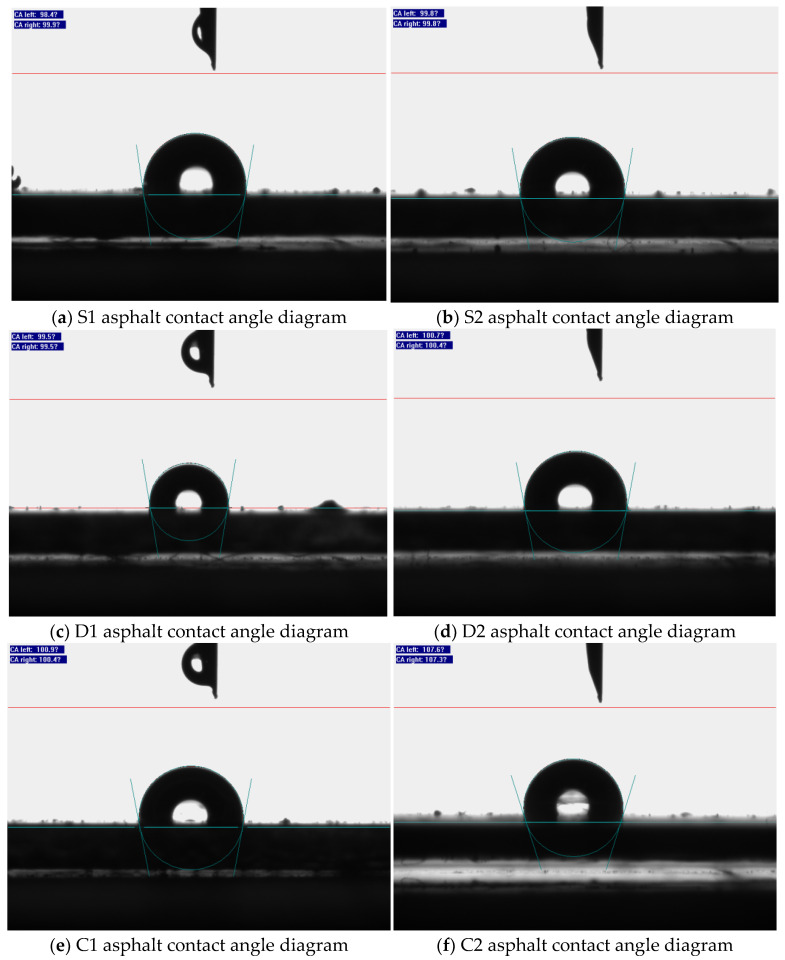
Contact angle diagram.

The contact angle is the tangent of the gas–liquid interface where the three phases of gas, liquid, and solid meet [34,35,36]. The contact angles (between the distilled water and the asphalt surface) of the two asphalts before and after different degrees of aging are shown in Figure 8. A contact angle θ > 90° indicates that the solid surface is more hydrophobic, i.e., the liquid does not easily wet the solid and has more mobility on its surface. The specific values of the contact angle for each group of tests are characterized in the pictures.

As shown in the figure, the contact angle between both asphalts and distilled water gradually increased as the degree of aging intensified, indicating that the aging process enhanced the hydrophobicity of the asphalt [37,38]. Moreover, it can be seen from the figure that the addition of Graphene oxide can increase the contact angle between the water molecules and the asphalt coating, i.e., the asphalt samples with Graphene oxide are more hydrophobic and less sensitive to water, and this improvement is more obvious as the aging process progresses.

The contact angle test shows that the addition of Graphene oxide to Polyurethane-SBS modified asphalt can reduce the water sensitivity of asphalt and reduce the effect of water corrosion, especially for the aging asphalt to improve the effect significantly.

### 3.4. Fluorescence Microscopy Test

**Figure 9:**
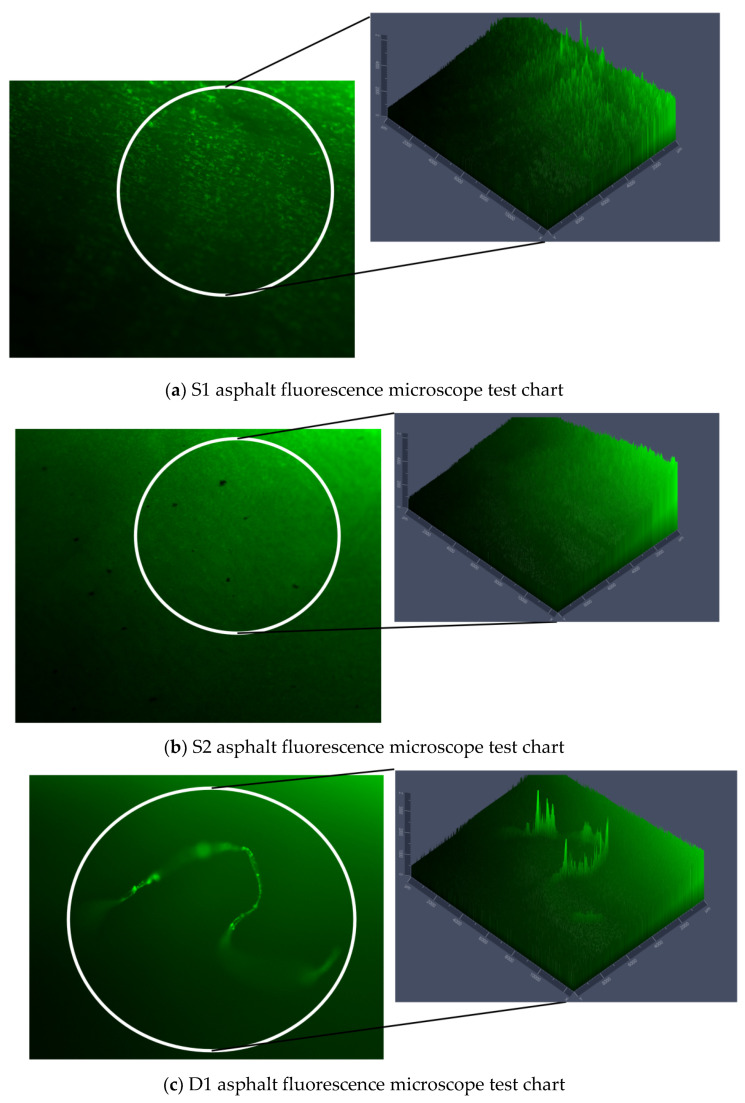

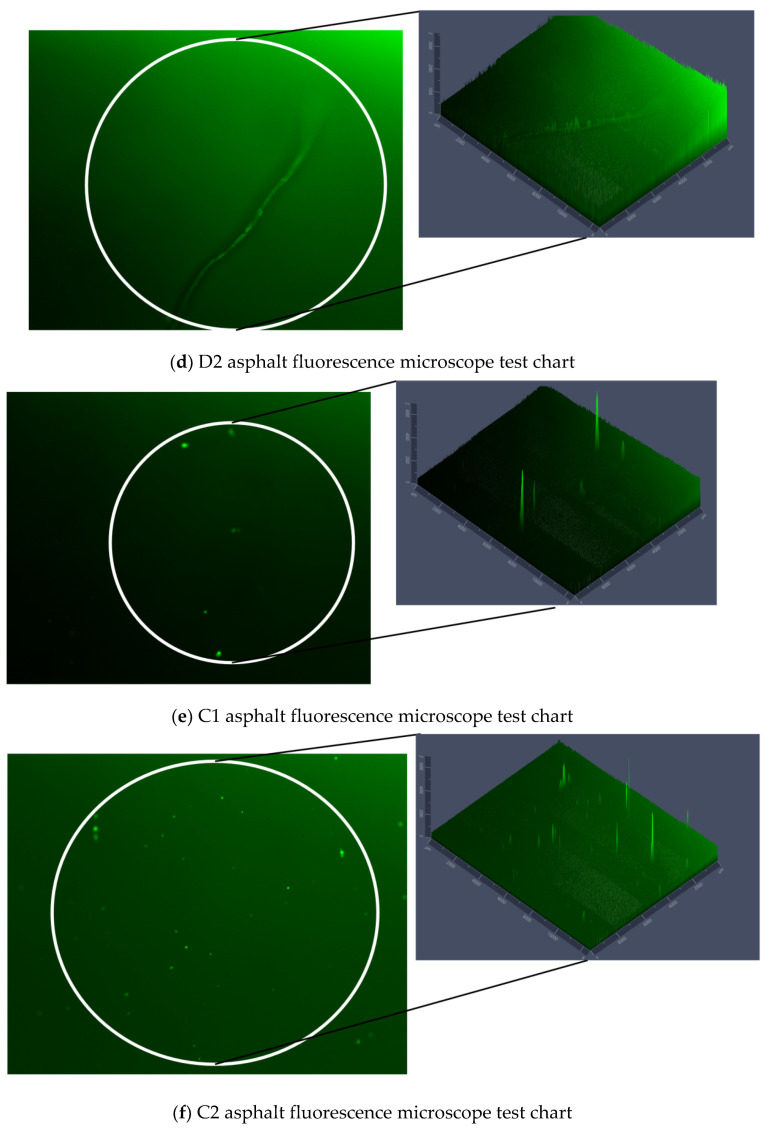
Fluorescence microscope observation plan and 2.5D view test chart for two asphalts with different aging levels.

Fluorescence microscopy allows the observation of the surface morphology and internal structure of objects using fluorescence beams [39]. In order to observe the effect of Graphene oxide on Polyurethane-SBS modified asphalt at different aging stages [5,40], Polyurethane-SBS modified asphalt and Graphene oxide-Polyurethane-SBS modified asphalt at different aging stages was tested by fluorescence microscopy. The tests are shown in Figure 9.

From Figure 9a,b, it can be seen that the synergistic effect of Polyurethane and SBS modifier can form irregular mesh structure in asphalt, and the addition of Graphene oxide can make the phase structure more dense and stable, and the modifier can be dispersed more uniformly in asphalt, so that it can withstand the disturbance of external unfavorable factors to a certain extent and has better stability performance. As can be seen in Figure 9c,d, the asphalt subjected to short-term aging suffers a certain degree of damage to the mesh structure, and the modifier particles have a tendency to agglomerate, but the addition of Graphene oxide can slow down this agglomeration. As seen in the 2.5D peak plot, with the asphalt with Graphene oxide added, the modifier particles are still more uniformly dispersed. From Figure 9e,f, it can be seen that the fluorescent particle shapes of the two modified asphalts become smaller and the mesh structure is completely destroyed as the degree of thermal aging continues to deepen, but the asphalt with the addition of Graphene oxide has a relatively larger distribution of fluorescent points and more uniform dispersion, and the coverage of the small molecular structure formed by the oxidative decomposition of the asphalt per unit area by thermal aging is higher.

From the fluorescence microscopy test, it can be seen that the addition of Graphene oxide to Polyurethane-SBS modified asphalt can make the asphalt phase structure more dense and stable and enhance the aging resistance of the asphalt matrix.

## 4. Discussion

In this study, in order to clarify the effect of the new nanomaterial Graphene oxide on the performance of Polyurethane-SBS modified asphalt and asphalt mixes under the effect of thermal aging, a series of tests were conducted on Graphene oxide-Polyurethane-SBS modified asphalt and Polyurethane-SBS modified asphalt and two asphalt mixes, and the conclusions obtained are summarized as follows.

1. The addition of Graphene oxide can solidify the formation of irregular mesh structure in asphalt under the synergistic effect of Polyurethane and SBS modifier, making the asphalt phase structure more dense and stable so that it can withstand a certain degree of external adverse factors of perturbation, with better stability performance.
2. Graphene oxide modifiers can reduce the sensitivity of asphalt molecules to water, making asphalt with Graphene oxide more hydrophobic and more effective in improving asphalt after aging.
3. The addition of Graphene oxide improves the fracture resistance of asphalt mixtures as well as the cracking performance after aging, but Graphene oxide-Polyurethane-SBS modified asphalt mixture pavements require proper maintenance work.
4. The introduction of Opencv images can more clearly express the improvement effect of Graphene oxide on the fracture and aging resistance of asphalt mixes.

The results of the study showed that the addition of Graphene oxide improved the fracture resistance, water affinity properties, and aging resistance of asphalt and its mixes compared with Polyurethane-SBS modified asphalt. Compared with the current road asphalt, its anti-aging performance has been significantly improved. Therefore, the new nano-material graphene oxide added to Polyurethane-SBS modified asphalt is meaningful and feasible, graphene oxide-polyurethane-sbs composite modified asphalt as a new nano-material modified asphalt, is stronger in ultraviolet light than asphalt prone to aging. Its application to the field of road projects is very promising.

## Data Availability

The data of this study have been included in the manuscript.
